# Risk prediction models for postoperative delirium in elderly patients with hip fracture: a systematic review

**DOI:** 10.3389/fmed.2023.1226473

**Published:** 2023-09-15

**Authors:** Yaqi Hua, Yi Yuan, Xin Wang, Liping Liu, Jianting Zhu, Dongying Li, Ping Tu

**Affiliations:** ^1^Department of Critical Care Medicine, The Second Affiliated Hospital of Nanchang University, Nanchang, Jiangxi, China; ^2^School of Nursing, Nanchang University, Nanchang, Jiangxi, China; ^3^School of Nursing, University of South China, Hengyang, Hunan, China; ^4^Department of Postanesthesia Care Unit, The Second Affiliated Hospital of Nanchang University, Nanchang, Jiangxi, China

**Keywords:** hip fracture, postoperative delirium, prediction, models, systematic review

## Abstract

**Objectives:**

To systematically evaluate the risk prediction models for postoperative delirium in older adult hip fracture patients.

**Methods:**

Risk prediction models for postoperative delirium in older adult hip fracture patients were collected from the Cochrane Library, PubMed, Web of Science, and Ovid via the internet, covering studies from the establishment of the databases to March 15, 2023. Two researchers independently screened the literature, extracted data, and used Stata 13.0 for meta-analysis of predictive factors and the Prediction Model Risk of Bias Assessment Tool (PROBAST) to evaluate the risk prediction models for postoperative delirium in older adult hip fracture patients, evaluated the predictive performance.

**Results:**

This analysis included eight studies. Six studies used internal validation to assess the predictive models, while one combined both internal and external validation. The Area Under Curve (AUC) for the models ranged from 0.67 to 0.79. The most common predictors were preoperative dementia or dementia history (OR = 3.123, 95% CI 2.108–4.626, *p* < 0.001), American Society of Anesthesiologists (ASA) classification (OR = 2.343, 95% CI 1.146–4.789, *p* < 0.05), and age (OR = 1.615, 95% CI 1.387–1.880, *p* < 0.001). This meta-analysis shows that these were independent risk factors for postoperative delirium in older adult patients with hip fracture.

**Conclusion:**

Research on the risk prediction models for postoperative delirium in older adult hip fracture patients is still in the developmental stage. The predictive performance of some of the established models achieve expectation and the applicable risk of all models is low, but there are also problems such as high risk of bias and lack of external validation. Medical professionals should select existing models and validate and optimize them with large samples from multiple centers according to their actual situation. It is more recommended to carry out a large sample of prospective studies to build prediction models.

**Systematic review registration:**

The protocol for this systematic review was published in the International Prospective Register of Systematic Reviews (PROSPERO) under the registered number CRD42022365258.

## 1. Introduction

As the global population continues to age, the incidence of hip fractures and their associated economic burden is rapidly increasing (1). According to Cooper et al., 1.6 million hip fractures occurred among the 9 million osteoporotic fracture patients worldwide in 2000, and they predicted that 6.3 million hip fractures would occur worldwide in 2050 (2). The Asian Federation of Osteoporosis Society (AFOS) reports an increase in the number of hip fractures in Asia from 1.12 million in 2018 to 2.56 million in 2050 (3). Currently, surgical treatment is the primary means of treating hip fractures, and the American Academy of Orthopaedic Surgeons (AAOS) emphasizes the importance of performing emergency surgery for older adult hip fractures within 24–48 h to provide better functional outcomes for patients (4). Older adult patients are at a higher risk of postoperative complications, and postoperative delirium (POD) is one of the most common complications among them. The incidence of postoperative delirium in older adult hip fracture patients is approximately 50% (5, 6). Postoperative delirium is an acute fluctuating dysfunction of the patient’s central nervous system in the postoperative period, mainly manifested as a decline in consciousness and cognitive function, and usually occurs between 24 and 72 h after surgery (7). Postoperative delirium can cause a series of adverse prognoses, including increased patient mortality, prolonged hospitalization, and increased economic burden on families and society (6). Therefore, early recognition and active treatment of postoperative delirium are crucial. Many scholars worldwide have developed single-center or multi-country models using various research designs to predict the risk of postoperative delirium in older adult hip fracture patients. The present study aims to comprehensively retrieve studies on the postoperative delirium risk prediction models for older adult hip fracture patients, and to systematically summarize and compare them from the perspectives of the basic characteristics, construction methods, methodological quality, prediction effectiveness, and prediction factors of the models. Our study provides a theoretical basis for the construction and application of postoperative delirium risk prediction models for older adult hip fracture patients.

## 2. Methods and analysis

The protocol for this systematic review was published in the International Prospective Register of Systematic Reviews (PROSPERO) under the registered number CRD42022365258. This systematic review was reported according to the Preferred Reporting Items for Systematic Reviews and Meta-Analyses (PRISMA) checklist.

### 2.1. Patient and public involvement

Patients and the public were not involved in the design or conduct of this systematic review.

### 2.2. Search strategy

Articles on risk prediction models for postoperative delirium in older adult patients with hip fractures were searched until March 15, 2023, using the following databases: the Cochrane Library, PubMed, Web of Science, and Ovid. The following terms are used: “hip fracture” and “delirium.” Our complete search string for PubMed was “(hip fracture OR trochanteric fracture OR subtrochanteric fracture OR hip joint implantation OR hip replacement OR hip arthroplasty) AND (delirium OR disturbance of consciousness OR cognitive impairment OR excitement OR excitement OR POD OR POCD).” The search is limited to Titles/Abstract and the references of all original articles were screened (See Appendix 1). The language of the articles was English.

### 2.3. Eligibility criteria

Articles meeting the following criteria were included: (1) Study designs, cohort study or case–control study; (2) Populations, older adult hip fracture patients with an age over 60 years; (3) Outcome, postoperative delirium; and (4) the research content, tools, and methods used for the construction of the risk prediction model were given in detail, and internal or external validation was carried out after the establishment of the prediction model. We excluded articles where (1) the development process or method for establishing the model was not described; (2) the model’s predictors cannot be widely evaluated or accurately measured in clinical practice; (3) full-text of the article was not available; and (4) Repeated publications.

### 2.4. Literature screening and data extraction

Two researchers independently screened the literature, extracted the data, and cross-checked the data. In the case of disagreement, they consulted a third party. For literature screening, we first read the title and abstract, and after excluding irrelevant literature, we further read the full text to determine inclusion. The extracted data included the first author, time of publication, country, research type, participants, modeling sample size and outcome events, modeling methods and verification model method, criteria for POD (Postoperative Delirium), model performance including Area Under Curve (ACU) and calibration methods, number and names of predictive factors, and risk factor assignment/risk stratification method.

### 2.5. Statistical analysis

The meta-analysis used Stata (version 13.0) to extract research data and generate the forest map. In our meta-analysis, the Odds Ratio (OR) and corresponding 95% Confidence Interval (CI) were combined to explore the relationship between the risk factors and POD in older adult patients with hip fracture. We detected heterogeneity using the Q test. When *p* < 0.1 or I^2^ > 50%, the random effect model is selected; When *p* > 0.1 and I^2^ < 50%, select the fixed effect model. After a combined analysis, it was considered statistically significant when *p* < 0.05. A sensitivity analysis was conducted to detect sources of heterogeneity by removing each study from the meta-analysis independently. Potential publication bias was judged by Begg’s test and Egger’s test; *p* < 0.05 was considered significant. If there was a potential bias, the trim-and-fill method was used to reassess.

### 2.6. Literature quality evaluation

Quality was assessed using the Newcastle-Ottawa scale (NOS), which includes 3 major dimensions: selection, comparability, and exposure. The evaluation was scored out of 9, with a score of ≥7 being good-quality literature and < 7 being inferior-quality literature.

### 2.7. Bias risk assessment

The two researchers (HYQ and YY) independently assessed the risk of bias in the selected studies using PROBAST, and a third party (TP) determined the difference. The PROBAST, which was developed by Wolff and his team in 2019, includes a risk of bias assessment and an applicability evaluation (8). PROBAST is organized into four domains, including participants, predictors, outcomes, and analysis. Based on the evaluation results of each domain, the risk of bias and applicability of the prediction model were obtained (9).

### 2.8. Predictive performance

Predictive performance is mainly evaluated from the perspectives of discrimination and calibration. The discrimination is measured by the AUC (AUC ≥ 0.7 indicates good model discrimination), among which we believe that the AUC of external testing is more representative than the AUC of internal testing. The calibration is evaluated through the Hosmer-Lemeshow test (when the Hosmer-Lemeshow test *p* > 0.05, it indicates good model fit; otherwise, it is considered poor model fit) and the calibration plots (when the calibration slope is close to 1, it is considered that the model fits well).

## 3. Results

### 3.1. The screening process and results

**Figure 1:**
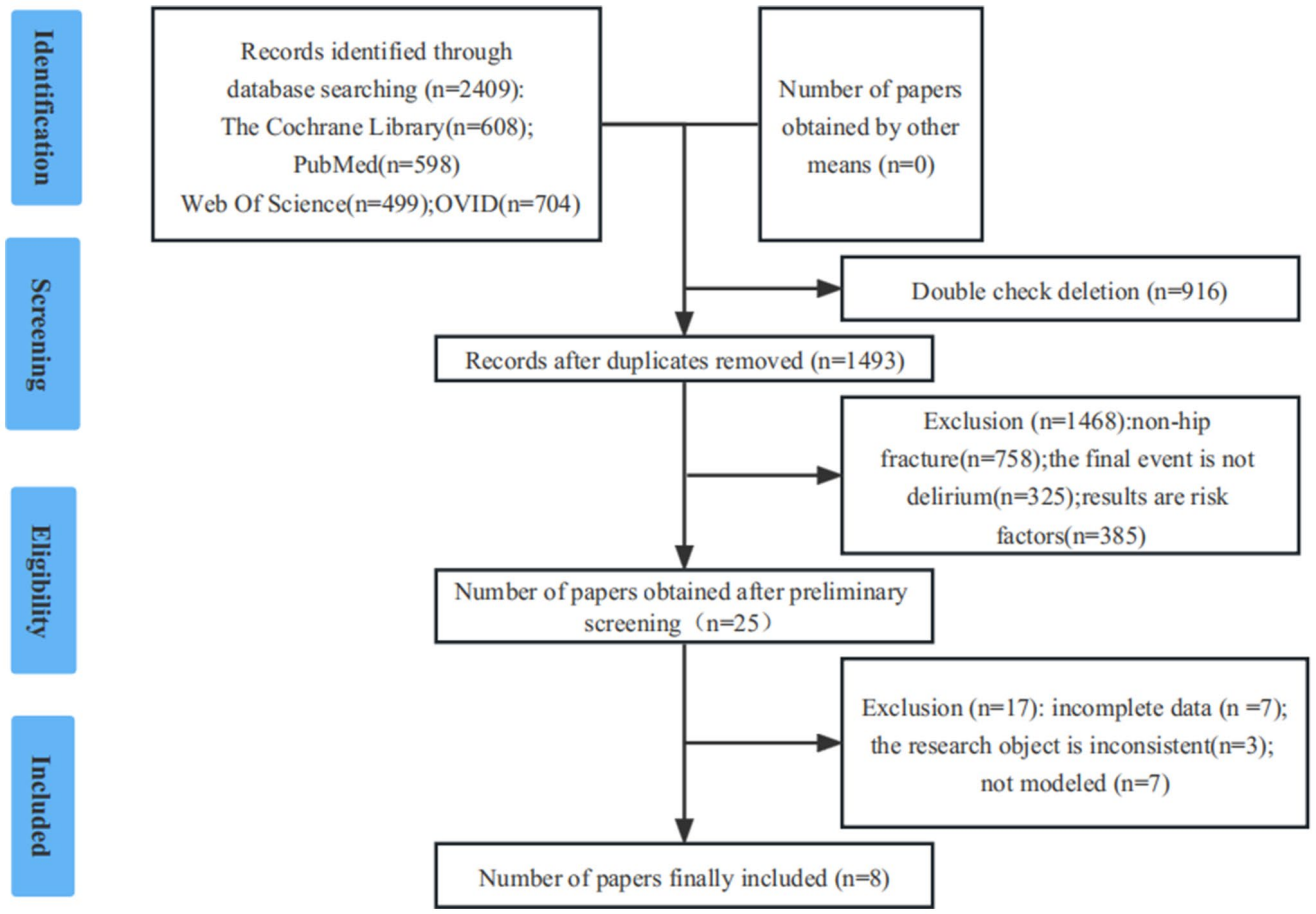
PRISMA flow diagram of study selection process.

Initially, the researchers identified 2,409 studies. After screening, the final analysis included 8 studies (7, 10–16). In the evaluation of literature quality, 8 (≥7 points) were of high-quality. The details were provided in Figure 1.

### 3.2. General information on included studies

A total of eight risk prediction models for postoperative delirium in older adult hip fracture patients were included, including three studies conducted in the United States of America (USA), four in China, and one in Australia and New Zealand (7, 10–16). In terms of research type, one was a prospective cohort study (10), one was a case–control study (16), and the other six were retrospective cohort studies (10). The earliest risk prediction model was published in 2006 (10), and six articles were published in the last 3 years (7, 11–14, 16). Five studies (10, 12, 13, 15, 16) defined the participants as patients with hip fracture or proximal femur fracture aged 65 years or older, and the other three studies (7, 11, 14) defined age as 60 years or older, resulting in a high level of homogeneity (Table 1).

**Table 1:** Basic characteristics included studies.

Study	Country	Research type	Fracture site	Age range (years)	Nos
Goldenberg et al. (10)	United States	Prospective cohort study	Proximal femur	≥65	8
Kim et al. (11)	United States	Retrospective cohort study	Hip bone	≥60	7
Oberai et al. (12)	Australia and New Zealand	Retrospective cohort study	Proximal femur	≥65	7
Oosterhoff et al. (7)	United States	Retrospective cohort study	Hip bone	≥60	7
Wang et al. (13)	China	Retrospective cohort study	Hip bone	≥65	8
Yang et al. (14)	China	Retrospective cohort study	Hip bone	≥60	7
Zhang et al. (15)	China	Retrospective cohort study	Proximal femur	≥65	7
Zhao et al. (16)	China	Case–control study	Hip bone	≥65	7

### 3.3. Model modeling and validation methods

In the included models, the modeling sample size was 77 ~ 22,563, and the incidence of delirium was 13.04% ~ 48.05%. In terms of modeling methods, three studies used single factor analysis to select the factors related to postoperative delirium in older adult hip fracture patients, and then used logistic regression to select independent predictive factors and modeling (10, 12, 15); one study used Lasso regression and logistic regression modeling (14); there are two studies using a recursive random forest (RF) algorithm to identify variables that may be relevant; finally, the Machine learning (ML) algorithm constructs the model (7, 16); and two studies used stepwise regression analysis to obtain the prediction model (11, 13). As for the method of validating the model, one study used internal validation and external validation (14), while five studies only used internal validation (7, 11, 12, 15, 16) (Table 2).

**Table 2:** Model effectiveness evaluation included studies.

Study	Modeling sample size		Modeling method	Verification model method	Criteria for POD	Model performance	
	Total	Outcome events				AUC (Modeling/ Verification)	Calibration test method
Goldenberg et al. (10)	77	37	Logistic regression	–	CAM	−/−	–
Kim et al. (11)	6,210	1816	Logistic regression	Internal	Delirium Chart Determination Developed by ACS-NSQIP	0.77/0.77	Calibration plots
Oberai et al. (12)	3,336	1,326	Logistic regression	Internal	CAM, 4AT, CAM-ICU, 3D-CAM	0.74/0.75	H-L test
Oosterhoff et al. (7)	22,563	–	Machine learning (SGM, RF, SVM, NN, PLR)	Internal	Delirium Chart Determination Developed by ACS-NSQIP	0.79/−	Calibration plots
Wang et al. (13)	272	52	Logistic regression	–	CAM	−/−	–
Yang et al. (14)	230	30	Logistic regression	Internal+ External	CAM	0.79/0.84	H-L test
Zhang et al. (15)	825	118	Logistic regression	Internal	DSM-V	0.67/−	H-L test and calibration plots
Zhao et al. (16)	245	30	Machine learning (RF, XGBoost, SVM, MLP)	Internal	CAM	0.78/−	–

“–” means not stated in the paper. AUC, area under curve, CAM, the confusion assessment method; ACS-NSQIP, American college of surgeons-national surgical quality improvement program; 4AT, 4‘A’s Test; CAM-ICU, the confusion assessment method for the intensive care unit; 3D-CAM, 3-min diagnostic interview for CAM; H-L test, Hosmer-Lemeshow test; SCM, stochastic gradient boosting; RF, random-forest; SVM, support vector machine; NN=neural network; PLR, elastic-net penalized logistic regression; DSM-V, diagnostic and statistical manual of mental disorders, 5th edition; XGBoost, eXtreme gradient boosting; MLP, multilayer perception.

### 3.4. Predictors and assignment

Of the eight included studies, at most 9 predictors were included (11), and at least 3 predictors were included (14). In the present systematic review, the most common predictors of postoperative delirium in older adult hip fracture patients were preoperative dementia or history of dementia (*n* = 5), ASA classification (*n* = 4), and age (*n* = 3). In terms of the risk factor assignment, three studies assigned the value of prediction factors by OR values of logistic regression, and the scores were the sum of the scores of each prediction factor for final risk judgments (11, 13, 15). Based on β coefficient of logistic regression, three other studies assigned weight to each predictor (10, 12, 14). The last two studies generated specific delirium prediction models based on machine learning to determine the weights of prediction factors, and then predicted the probability of delirium occurrence (7, 16), as detailed in Table 3.

**Table 3:** Predictors and stratification methods included in the study.

Study	Number of factors	Predictors	Risk factor assignment/Risk stratification method
Goldenberg et al. (10)	6	Age>81, medication history, ST < 20 points, MMSE<24 points, Alb<3.5 g/dL and Hct < 33 (ST:The set test as an aid to the detection of dementia in old people)	Through the β coefficient gives the delirium probability p formula, which is: *p* = 1/{1 + exponent (−a)}.Among them, a = −7.6 + [multiple medications× 3.5] + [ST × 2.6] + [MMSE × 1.9] + [Alb × 1.8] + [Hct × 1.6] + [age × 0.6]。 According to β coefficient is assigned to each factor and added to get the total score. The total score range is 0–14, of which 0–3 is the low-risk group; 4–6 moderate risk group; 7–10: high-risk group; 11–14: a very high-risk group
Kim et al. (11)	9	Preoperative delirium, preoperative dementia, age, medical co-management, ASA ≥ III，functional dependence, smoking, systemic inflammatory response syndrome/Sepsis/Septic shock, and preoperative use of mobility aid	The odds ratio (OR) in the logistic regression model is rounded and added with scores. The total score is from 0 to 20. The risk of POD varies from 4.5 to 92.0%.
Oberai et al. (12)	7	Age>80, male, absent pre-operative cognitive assessment, impaired pre-operative cognitive state, prior impaired cognition or known dementia, surgery delay and mobilization day 1 post-surgery	The β coefficient in the logistic regression model multiply by 10 and round to get an integer. Add up to get the total score. Delirium risk score < 10, 10–19, 20–29, 30–39, 40 +, corresponding risk incidence was 14.2, 30.6, 53.8, 75.5 and 89.1%, respectively.
Oosterhoff et al. (7)	6	Age ≥ 90, ASA ≥ II, functional status, preoperative dementia, preoperative delirium, preoperative need for mobility-aid	No specific description of risk factor assignment/risk stratification method. Tool location: https://sorg-apps.shinyapps.io/hipfxdelirium/
Wang et al. (13)	6	Drinking history (> 3/ week), Lac >2 mmol/L, postoperative VAS > 3, ASA > II, preoperative diabetes, application of the bispectrality index	The odds ratio (OR) in the logistic regression model was used to assign values to each factor. The total score range is −1 ~ 8 points, and the corresponding POD incidence rates are 0, 0, 1.72, 9.80, 14.29, 26.47, 61.54, 100, 100 and 100% respectively
Yang et al. (14)	3	Dementia, chronic obstructive pulmonary disease, and Alb	The risk factors are scored according to the β coefficients in the logistic regression analysis, and visualized using nomograms.
Zhang et al. (15)	5	Preoperative cognitive impairment, Complications ≥ two, ASA ≥ III, transfusion >2 units of red blood cell, and intensive care	The odd ratio (OR) in the logistic regression model is used to assign values to each factor and the nomogram is used for visualization. The total score ranges from 0–24, and the higher the score, the greater the risk.
Zhao et al. (16)	6	Preparation time, frailty index, uses of vasopressors during the surgery, dementia/history of stroke, duration of surgery and type of anesthesia	The machine learning model assigns the correlation coefficient of risk factors, but does not explain the method of risk factor assignment /risk stratification.

ST, the set test; MMSE, Mini-mental state examination; Alb, albumin; Hct, red blood cell specific volume; ASA, American society of Anesthesiologists physical status classification system; Lac, the perioperative lactic acid level; VAS, visual analogue scale.

### 3.5. Meta-analysis for risk factors

**Figure 2:**
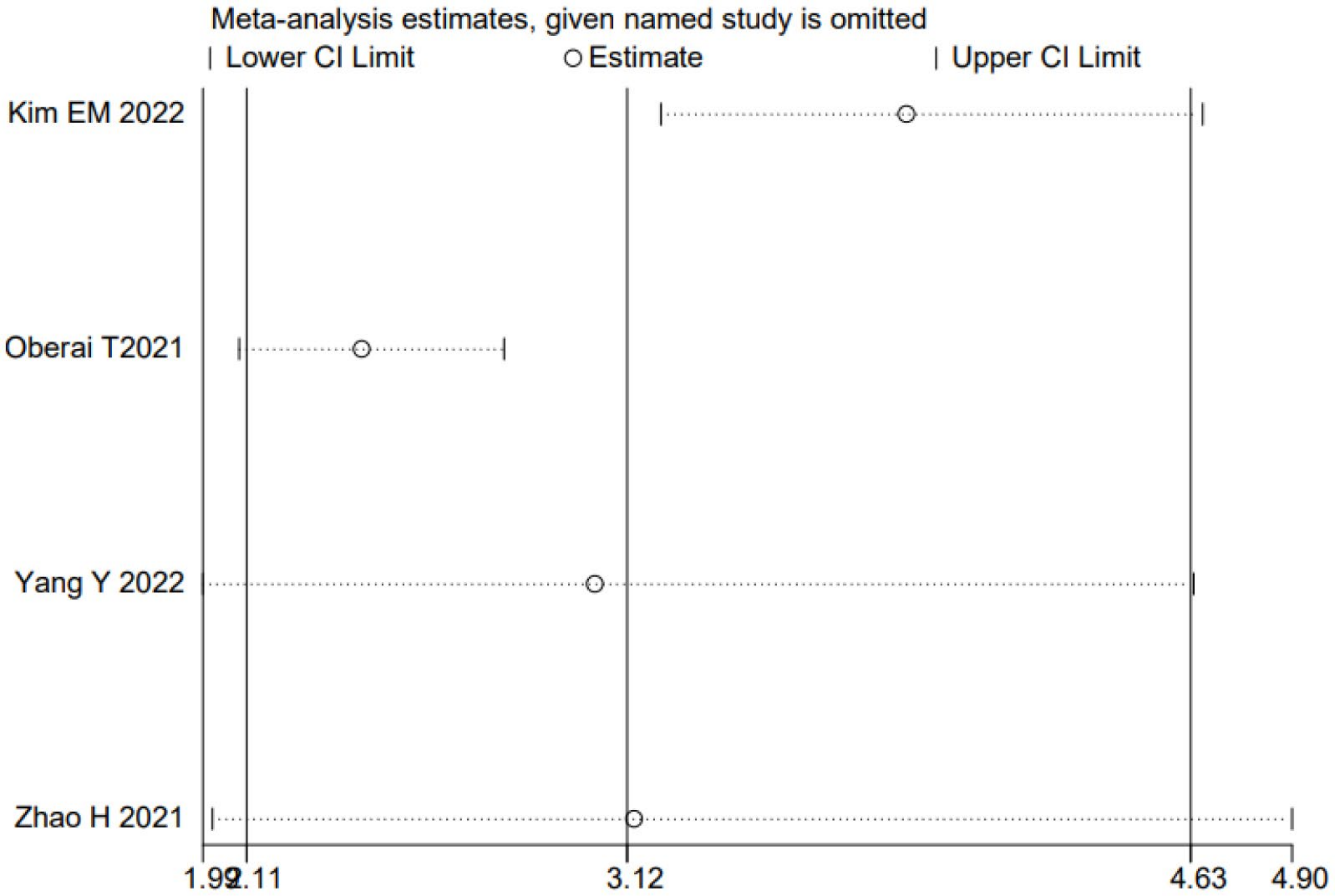
Sensitivity analysis for the association between dementia and POD.

**Figure 3:**
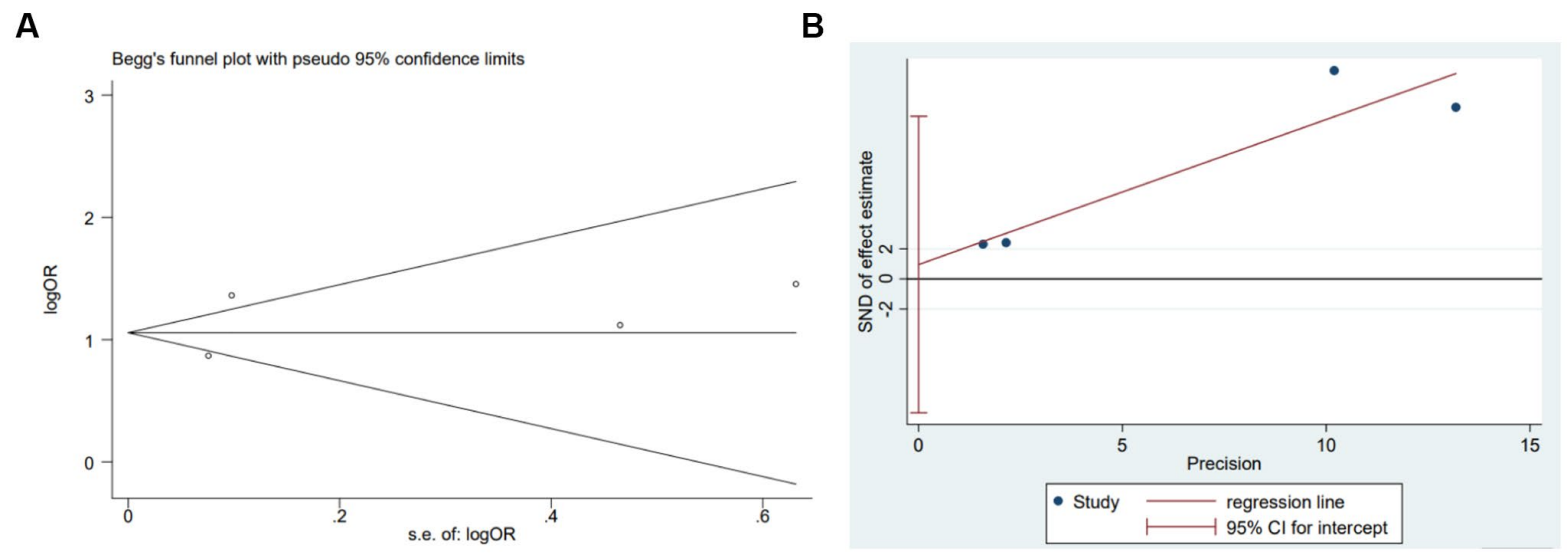
Plots for publication bias test in meta-analysis for the association between dementia and POD. **(A)** Begg’s funnel plot; **(B)** Egger’s publication bias plot.

We performed a meta-analysis for preoperative dementia or history of dementia, ASA classification, and age. Due to the inability to extract the required data from literature such as Oosterhoff JHF (7), a meta-analysis was conducted on the remaining studies after exclusion. The results indicated that preoperative dementia or history of dementia, ASA classification, and age were independent risk factors for postoperative delirium in older adult patients with hip fracture. The results are presented in Table 4. As an example, a sensitivity analysis was drawn for dementia. We further explored the source of heterogeneity by removing each study from the meta-analysis independently. The results showed that ignoring any of the enrolled studies did not significantly change the effect of the dementia on the combined meta-analysis for POD. That indicated that the overall results were stable and reliable (Figure 2). In the meta-analysis for dementia, Begg’s test (*p* = 0.734) and Egger’s test (*p* = 0.716) determined no significant publication bias (Figures 3A,B).

**Table 4:** The meta-analysis for risk factors.

Factors	No. of studies	Effects model	OR (95%CI)	*P*	Heterogeneity	
					*I*^2^ (%)	*P*_Q_
Dementia	4 (11, 12, 14, 16)	REM	3.123 (2.108–4.626)	<0.001	81.6	0.001
ASA classification	3 (11, 13, 15)	REM	2.343 (1.146–4.789)	<0.05	85.8	0.001
Age	2 (10, 12)	FEM	1.615 (1.387–1.880)	<0.001	43.8	0.0.182

FEM, fixed-effects model; REM, random-effects model.

### 3.6. Methodological quality evaluation

In the eight included articles, five studies were at high risk of bias in the bias risk assessment (10, 12, 13, 15, 16). The high-risk areas were mainly participants and statistical analysis. Two studies (11, 14) were at unclear risk, and the remaining one (7) was at low bias risk. In terms of applicability evaluation, six studies were low risk of applicability (7, 10–14), and two studies (15, 16) were unclear risk of applicability levels (Table 5).

**Table 5:** Risk of bias assessment results included in the model (PROBAST).

Study	Risk of bias assessment				Applicability evaluation			Total
	Participants	Predictors	Outcome	Analysis	Participants	Predictors	Outcome	
Goldenberg et al. (10)	1	1	1	3	1	1	1	3/1
Kim et al. (11)	1	1	1	2	1	1	1	2/1
Oberai et al. (12)	1	1	1	3	1	1	1	3/1
Oosterhoff et al. (7)	1	1	1	1	1	1	1	1/1
Wang et al. (13)	3	1	2	3	1	1	1	3/1
Yang et al. (14)	2	1	1	2	1	1	1	2/1
Zhang et al. (15)	3	1	2	3	1	1	2	3/2
Zhao et al. (16)	3	1	2	3	1	1	2	3/2

1 = low risk; 2 = unclear risk; 3 = high risk; total means risk of bias assessment/applicability evaluation.

### 3.7. Predictive performance evaluation

We evaluated the performance of the model from the perspectives of discrimination and calibration. In terms of discrimination, Zhang et al. (15) reported that the AUC was only 0.67, which indicates that the model has poor discrimination; the modeling AUC in both articles (7, 16) is greater than 0.7; Kim et al. and Oberai et al. (11, 12) reported that both the modeling and internal validation AUC were greater than 0.7; and the model developed by Yang et al. (14) performs best in discrimination, with AUC > 0.7 for both modeling and external validation. In terms of calibration, three articles (12, 14, 15) used the Hosmer-Lemeshow test, but the model developed by Oberai et al. (12) exhibited a *p* < 0.05 test result, indicating poor model fitting; three articles (7, 11, 15) used calibration plots, with calibration slopes close to 1 and indicated good calibration. The model of Zhang et al. (15) showed good calibration on both methods.

## 4. Discussion

In general, researchers are still in the developmental stage of studying risk prediction models for postoperative delirium in older adult hip fracture patients. The research spans a large period of time, and the number of studies is far less than that of risk factors. Researchers have concentrated the existing studies in America, China, Australia, and New Zealand, and most of the models have not been utilized in clinical practice since their establishment.

### 4.1. Prediction factor analysis

These eight prediction models in the collected studies include many prediction factors, such as socio-demographic information, medical information, scale test results, and clinical information, which can be obtained through simple and rapid inquiry or evaluation. Although the number and type of prediction factors in each model differed, there are some commonalities. Among them, a history of preoperative dementia or dementia history, ASA classification, and age were high correlated with postoperative delirium in older adult hip fracture patients, and meta-analysis suggests that they are independent risk factors, which is highly consistent with many other studies exploring the risk factors of postoperative delirium in older adult hip fracture patients (17–20). A history of preoperative dementia or dementia is a predictive factor of concern, and preoperative dementia patients are a special subpopulation (17). As a cognitive dysfunction, although there is no international consensus on the effect of preoperative dementia or dementia history on POD, it has been proven to be correlated with postoperative delirium (21–23). Rong et al. conducted a meta-analysis including 22 articles on the risk factors of postoperative delirium after knee and/or hip replacement, of which 16 articles were on older adult patients with hip replacement (21). They found that dementia is a risk factor for postoperative delirium (21). Lee et al. conducted a prospective cohort study on older adult hip fracture patients and found that the incidence of postoperative delirium in patients with preoperative dementia or dementia history was 2.1 times higher than that in the control group (23). A history of preoperative dementia or dementia may cause brain metabolic disorders and polyamine pathway disorders, which may contribute to postoperative delirium (24). Change in polyamine level caused by the imbalance in the polyamine pathway will result in abnormal ion channel and ion glutamate receptors, followed by electrolyte disorder. At the same time, electrolyte disorder can lead to microcirculation disorder, which plays a particularly important role in the occurrence of postoperative delirium (25–27).

The ASA classification is used to assess the general disease status and overall health status of patients and is one of the most valuable methods for preoperative determination of surgical and anesthetic risk (28). Although the ASA classification was originally designed as an anesthetic risk assessment system, it is now widely used to predict perioperative risk and mortality (29, 30). Hackett et al. also believed that the higher the ASA classification, the worse the overall health of patients, and the more significantly increased postoperative complications (31). In addition, ASA classification can be used as a risk factor for postoperative death (28), and also as an independent risk factor for postoperative delirium in older adult hip fracture patients (32). Therefore, for patients with high ASA classification, medical personnel should strengthen preoperative continuous monitoring, postoperative prevention and treatment, improve the compensatory ability of each organ, and effectively prevent postoperative delirium.

Age is recognized as an independent risk factor for postoperative delirium (33, 34). Studies have confirmed a correlation between age and postoperative delirium in older adult hip fracture patients (6, 17). Haynes et al. studied 18,754 older adult hip fracture patients and confirmed that age was an independent predictor of postoperative delirium (17). The reason may be that with the increasing age, degenerative changes in the brain parenchyma of older adult patients occur, such as aging of nerve cells, reduction of cerebral blood flow perfusion, and changes in the content of central neurotransmitters, among which the change in the central neurotransmitters content is an influential cause of delirium (35, 36). Due to the weakened function of important organs such as the heart, brain, and lungs, the compensatory ability of older adult patients is significantly reduced, leading to reduced tolerance to anesthesia and surgery. This can result in severe hemodynamic fluctuations, stimulating the body to release inflammatory factors. These inflammatory factors can induce inflammatory responses in the central nervous system, causing changes in the cognitive level of patients and even postoperative delirium (37–39).

### 4.2. Discussion on overall bias risk

The risk of bias in prediction models is closely related to the source of participants, definition and evaluation of prediction factors, classification and definition of outcomes, and statistical analysis. The present systematic review included eight articles, of which five studies had a high risk of bias (10, 12, 13, 15, 16), two studies had uncertain bias risk (11, 14), and one study had a low risk of bias (7). The main reasons behind this are: (1) risk of bias in data sources; (2) insufficient sample size; (3) unreasonable processing of independent variables; (4) defects in processing methods for missing data; (5) adoption of single factor analysis to screen prediction factors; (6) lack of performance evaluation of prediction models; and (7) failure to consider whether there are problems with model fitting. PROBAST points out that data from randomized controlled trials, registered data, prospective cohort studies, Nested case–control studies, or case-cohort studies are superior to retrospective cohort studies and traditional case–control studies (8). However, only one in the 8 selected studies comes from prospective cohort study (10). In terms of sample size, PROBAST requires that model development studies should have more than 20 events per variable (EPV) to avoid overfitting of the model; model validation studies should include at least 100 subjects with outcomes (40). Most studies fail to meet the requires in the sample size of modeling or model verification, which increases the risk that the prediction model may contain incorrect predictors or fails to include significant predictors (7, 10, 13–16). Regarding the processing methods of independent variables, two studies simply classified continuous variables into binary variables (10, 13), and one study transformed continuous variables into ≥2 category variables, leading to losing lots of useful information and even reducing the predictive power of the model (11). For the processing of missing data, two studies had no missing data (10, 15), one study used multiple imputation to deal with missing values (7), while the remaining studies directly excluded the inclusion of missing data and used complete data analysis (11–14, 16). The use of univariate analysis to screen predictors is a routine strategy in model development studies. Three studies used univariate analysis to select relevant factors, but researchers do not recommend it as a basis for screening predictive factors (10, 12, 15). In univariate analyses, models end up incorporating inappropriate predictors or rejecting valid predictors because of collinearity between independent variables (41). Thus, according to the guidelines of the Transparent Reporting of a Multivariate Prediction Model for Individual Prognosis or Diagnosis (TRIPOD), it is recommended to use the stepwise regression method or appropriately adjust the significance level during univariate analysis (42). In terms of model performance, only five studies reported both AUC and calibration, and used Hosmer-Lemeshow tests or calibration plots to describe the calibration (7, 11, 12, 14, 15). Among them, the *p* value obtained by the Hosmer-Lemeshow test cannot be used to quantify the model calibration (43). It is recommended to use or combine calibration plots to describe the calibration of the prediction model. Three studies used calibration plots (7, 11, 15), and one study used both methods (15). Model performance indicator tend to have optimistic biases due to overfitting or the selection of better thresholds. Therefore, internal verification through Self-service Sampling or cross validation is necessary. Six of the included studies conducted internal testing (7, 11, 12, 14–16), three of which used the randomized splitting method, an inefficiency testing method (11, 12, 14); two of which used a combination of randomized splitting and K-fold (7, 16); and the remining one adopted the Self-service Sampling (15). One study used both internal validation and external validation, and the AUC value for external validation was 0.84 (14). In terms of model applicability, only two studies were unclear about the risk (15, 16), and the other studies had good applicability (7, 10–14). The overall applicability of the eight studies was good.

### 4.3. Advantages and limitations

#### 4.3.1. Advantages

(1) The risk prediction models of postoperative delirium in older adult hip fracture patients published in recent years are systematically integrated, and the participants, modeling methods, model performance, predictors, and scores are comprehensively introduced. (2) The PROBAST is used to evaluate the quality of published risk prediction models for postoperative delirium in older adult hip fracture patients, analyze the main problems in the construction of current prediction models, and provide references for later model development. (3) Quantitative analysis is applied to predictive factors via meta-analysis to enhance result credibility.

#### 4.3.2. Limitations

(1) The present study includes only English-language literature, and researchers acknowledge that some publication bias may exist. (2) There are differences in the study population and delirium assessment tools for the eight prediction models. (3) In terms of model validation, most of the included studies are only internally validated, and only one study is externally validated, but there is a lack of external validation with large samples and multiple centers, and further validation of the applicability and stability of the model is needed. (4) Some models are established earlier and model validation is not reported. Whether the model is applicable to current clinical practice needs to be further explored.

## 5. Conclusion

In summary, this study assessed eight risk prediction models for postoperative delirium in older adult hip fracture patients. Some models demonstrated good predictive performance, and all models showed low applicability risks. This is beneficial for early screening high-risk older adult hip fracture patients for postoperative delirium. However, due to the high overall risk of bias in the included studies, it is not appropriate to apply the prediction model directly to clinical practice. Medical professionals should select existing models in their own context and validate them with large samples from multiple centers to facilitate clinical practice. Moreover, prospective studies with large samples are recommended to build localized predictive models based on the TRIPOD and PROBAST.

## Data availability statement

The original contributions presented in the study are included in the article/Supplementary material, further inquiries can be directed to the corresponding author.

## Author contributions

YH and PT conceived of the idea, designed the study, searched the relevant database, and wrote the manuscript. YY and XW interpreted the data and other relevant information. LL and JZ analyzed the quality of each study and confirmed the analysis. DL and PT provided the examination for the methodology, and reviewed and revised the manuscript. All authors contributed to the article and approved the submitted version.

## Funding

This study was supported by Project of Education Department of Jiangxi Province (GJJ210183). No commercial entity was involved. Role of the Funding: The fund had no role in the design and conduct of the study; collection, management, analysis, and interpretation of the data; preparation, review, or approval of the manuscript; and decision to submit the manuscript for publication.

## Conflict of interest

The authors declare that the research was conducted in the absence of any commercial or financial relationships that could be construed as a potential conflict of interest.

## Publisher’s note

All claims expressed in this article are solely those of the authors and do not necessarily represent those of their affiliated organizations, or those of the publisher, the editors and the reviewers. Any product that may be evaluated in this article, or claim that may be made by its manufacturer, is not guaranteed or endorsed by the publisher.
