# Supplementary material for: Risk prediction models for postoperative delirium in elderly patients with hip fracture: a systematic review
Source: Front Med (Lausanne). 2023 Sep 15;10:1226473. doi: 10.3389/fmed.2023.1226473 (PMC10540206; doi:10.3389/fmed.2023.1226473)
Supplement: Supplementary file 1 [file Data_Sheet_1.docx]

**Search strategy**

Searches were performed using the following databases: The Cochrane Library, PubMed, Web of Science, and Ovid,

Full search strategy (or strategies):(Hip Fractures OR Fractures, Hip OR Trochanteric Fractures OR Fractures, Trochanteric OR Intertrochanteric Fractures OR Fractures, Intertrochanteric OR Subtrochanteric Fractures OR Fractures, Subtrochanteric OR Arthroplasty, Replacement, Hip OR Arthroplasties, Replacement, Hip OR Arthroplasty, Hip Replacement OR Hip Prosthesis Implantation OR Hip Prosthesis Implantations OR Implantation, Hip Prosthesis OR Prosthesis Implantation, Hip OR Hip Replacement Arthroplasty OR Replacement Arthroplasties, Hip OR Replacement Arthroplasty, Hip OR Arthroplasties, Hip Replacement OR Hip Replacement Arthroplasties OR Hip Replacement, Total OR Total Hip Replacement OR Total Hip Arthroplasty OR Arthroplasty, Total Hip OR Hip Arthroplasty, Total OR Total Hip Arthroplasties OR Replacement, Total Hip) AND (Delirium OR consciousness OR confusion OR postoperative delirium OR acute delirium OR disorientation OR cognitive impairment OR waking delirium OR Emergence Delirium OR Excitement OR Agitated OR Agitation OR POD OR POCD)

Taking PubMed as an example, it is limited to [Title/Abstract]. The specific search is as follows: Search: (Delirium[Title/Abstract] OR consciousness[Title/Abstract] OR confusion[Title/Abstract] OR postoperative delirium[Title/Abstract] OR acute delirium[Title/Abstract] OR disorientation[Title/Abstract] OR cognitive impairment[Title/Abstract] OR waking delirium[Title/Abstract] OR Emergence Delirium[Title/Abstract] OR Excitement[Title/Abstract] OR Agitated[Title/Abstract] OR Agitation[Title/Abstract] OR POD[Title/Abstract] OR POCD[Title/Abstract]) AND (Hip Fractures[Title/Abstract] OR Fractures, Hip[Title/Abstract] OR Trochanteric Fractures[Title/Abstract] OR Fractures, Trochanteric[Title/Abstract] OR Intertrochanteric Fractures[Title/Abstract] OR Fractures, Intertrochanteric[Title/Abstract] OR Subtrochanteric Fractures[Title/Abstract] OR Fractures, Subtrochanteric[Title/Abstract] OR Arthroplasty, Replacement, Hip[Title/Abstract] OR Arthroplasties, Replacement, Hip[Title/Abstract] OR Arthroplasty, Hip Replacement[Title/Abstract] OR Hip Prosthesis Implantation[Title/Abstract] OR Hip Prosthesis Implantations[Title/Abstract] OR Implantation, Hip Prosthesis[Title/Abstract] OR Prosthesis Implantation, Hip[Title/Abstract] OR Hip Replacement Arthroplasty[Title/Abstract] OR Replacement Arthroplasties, Hip[Title/Abstract] OR Replacement Arthroplasty, Hip[Title/Abstract] OR Arthroplasties, Hip Replacement[Title/Abstract] OR Hip Replacement Arthroplasties[Title/Abstract] OR Hip Replacement, Total[Title/Abstract] OR Total Hip Replacement[Title/Abstract] OR Total Hip Arthroplasty[Title/Abstract] OR Arthroplasty, Total Hip[Title/Abstract] OR Hip Arthroplasty, Total[Title/Abstract] OR Total Hip Arthroplasties[Title/Abstract] OR Replacement, Total Hip[Title/Abstract])
